# Anti-inflammatory and dry eye benefits of accelerated epi-off corneal cross-linking in pediatric keratoconus with allergic ocular surface disease and elevated MMP-9

**DOI:** 10.1186/s40662-025-00469-7

**Published:** 2026-01-02

**Authors:** Cosimo Mazzotta, Caterina Gagliano, Fabiana D’Esposito, Francesco Cappellani, Carlos Rocha De Lossada, Davide Borroni, Alessandro Meduri, Marco Ferrise

**Affiliations:** 1 Department of Medicine and Surgery, Ophthalmology Unit, Faculty of Medicine and Surgery, University Kore of Enna, Enna, Italy; 2 Siena Cross-Linking Center, Via Sandro Pertini 10, 53035 Monteriggioni, Siena Italy; 3 Regional University Hospital of Malaga, Málaga, Spain; 4 Department of Surgery, Ophthalmology Area, University of Seville, Seville, Spain; 5 Department of Ophthalmology, Riga Stradins University, Riga, Latvia; 6 Ophthalmology Clinic, Department of Biomedical Sciences, University of Messina, Messina, Italy; 7 Ferrise Eye Clinic, Lamezia Terme, Italy

**Keywords:** Corneal cross-linking, Dry eye disease, DED, Keratoconus, Ocular surface, Ocular surface inflammation, Tear film, Metalloproteinases, MMP-9

## Abstract

**Purpose:**

To assess the functional and ocular surface anti-inflammatory outcomes of epithelium-off accelerated corneal cross-linking (ACXL) in adolescents with progressive keratoconus associated with allergic ocular surface disease and dry eye disease (DED) characterized by elevated tear matrix metalloproteinase-9 (MMP-9) concentrations.

**Methods:**

Prospective interventional case series of 30 eyes from 15 patients (mean age 16.41 ± 2.36 years; Krumeich stage II) undergoing epi-off ACXL. Outcomes at baseline and 1, 3, 6, and 12 months included corrected distance visual acuity (CDVA), maximum keratometry (Kmax), minimum corneal thickness (MCT), computerized non-invasive tear break-up time (cBUT), Ocular Surface Disease Index (OSDI), and tear MMP-9 (point-of-care test). In vivo qualitative confocal microscopy (IVCM) investigation provided supportive imaging. Paired t-tests were used and results reported with 95% confidence intervals (CI).

**Results:**

CDVA improved to 0.09 logMAR at 12 months (≈ 0.81 decimal; 95% CI: 0.10–0.08 logMAR; *P* < 0.001). Kmax decreased from 55.00 to 53.75 D (95% CI: 53.55–53.95 D; Δ =  − 1.25 D; *P* < 0.001), indicating ectasia stabilization. cBUT increased from 10.11 to 14.41 s (95% CI: 14.11–14.71; *P* < 0.01). OSDI decreased to 12.15 (95% CI: 11.65–12.65). Tear MMP-9 levels diminished from 64.79 to 16.15 ng/mL (*P* < 0.0001) and the proportion < 38.6 ng/mL reached 86.7% of the study cohort at 12 months. IVCM documented disappearance of inflammatory infiltrates. No postoperative persistent adverse events occurred.

**Conclusions:**

Epi-off ACXL stabilized ectasia, improving visual and ocular surface outcomes, markedly lowering tear MMP-9 levels. Although exploratory, these findings are consistent with a potential ocular surface anti-inflammatory and neuromodulatory role of ACXL, meriting validation in studies involving inflammatory DED beyond keratoconus.

**Supplementary Information:**

The online version contains supplementary material available at 10.1186/s40662-025-00469-7.

## Background

Keratoconus (KC) is a degenerative corneal disorder that affects both the central and peripheral regions of the cornea. The disease primarily involves Bowman’s layer and the anterior-to-mid stroma [1, 2] leading to structural irregularities of the corneal surface that cause blurred vision, poor tear film quality and ocular irritation [3]. The prevalence of KC in the general population has been estimated to range between 0.2% and 2.3%, with considerable variability among different geographic regions [4–8].

KC is considered a multifactorial disease influenced by genetic predisposition [9] although increasing evidence highlights the role of inflammatory disease and elevated tear matrix metalloproteinases (MMPs) in both KC progression and dry eye disease (DED) [10]. Eye rubbing, common among KC patients, increases tear MMP levels, particularly MMP-9, which degrade corneal collagen and weaken the stromal structure, thereby promoting ectatic progression [11]. These inflammatory mediators also destabilize the tear film and irritate the ocular surface, perpetuating a vicious cycle of inflammation and dryness.

Environmental factors such as ultraviolet (UV) light exposure and recurrent trauma from habitual eye rubbing (reported in 40%–70% of patients) further accelerate disease progression [12]. Eye rubbing is often triggered by ocular discomfort, including burning, itching, and DED [13]. Moreover, chronic use of rigid gas-permeable contact lenses can induce inflammation and apoptosis of corneal and conjunctival epithelial cells due to mechanical stress [14–16].

Patients with KC frequently exhibit higher scores on the Ocular Surface Disease Index (OSDI), a validated questionnaire for assessing DED severity [17] reflecting the strong association between KC and ocular surface dysfunction. The irregular corneal shape of KC contributes to tear film instability and shortened tear break-up time (TBUT). This irregular surface, combined with frequent eye rubbing and inflammation, exacerbates evaporative dry eye [18]. Goblet cell apoptosis and reduced mucin production are also key factors contributing to DED in KC patients. Conjunctival impression cytology studies have demonstrated decreased goblet cell density and impaired mucin secretion, resulting in tear film instability and worsening dry eye symptoms [19].

Several studies have assessed DED parameters before and after corneal cross-linking (CXL), reporting significant reductions in OSDI scores and improvements in TBUT six months after treatment [20]. A transient increase in DED symptoms immediately after CXL has been observed, followed by gradual recovery over time [21]. Other studies have revealed a CXL-induced decrease in tear film MMP-9 concentrations [22].

Here, we aim to evaluate the functional and ocular surface anti-inflammatory outcomes of epithelium-off accelerated corneal cross-linking (ACXL) in adolescents with progressive KC associated with allergic ocular surface disease characterized by elevated tear MMP-9 concentrations and DED symptoms.

## Methods

### Study design and patient selection

This was a single-center, open-label, non-randomized prospective interventional pilot study evaluating epi-off ACXL in a case series of 30 eyes from 15 pediatric patients (mean age 16.41 ± 2.36 years) with progressive KC, ocular allergy, ocular surface inflammation and dry eye symptoms who tested positive for elevated tear MMP-9 levels. Two patients had vernal keratoconjunctivitis (VKC). Patients were enrolled between January and March 2024, and the last follow-up visit was completed in May 2025. The prospective study was approved by the Institutional Review Board of the Siena Cross-linking Center, Monteriggioni, Siena, Italy (IRB approval code: CXLMMP-9-P1) and adhered to the Declaration of Helsinki. Objective KC progression was required within 3–6 months prior to enrollment. Due to the higher risk of disease progression in pediatric patients [23], an untreated arm (not cross-linked) control group with medical therapy only was not approved. Written informed consent was obtained from caregivers (with minor assent as applicable). Postoperative outcomes were analyzed using clinical parameters, point-of-care inflammatory testing, and in vivo confocal microscopy (IVCM) qualitative investigation. Quantitative IVCM analysis was not planned in the original study design, as this examination was performed solely to provide supportive documentation for the laboratory findings and tomographic assessments, which were defined as the main outcome measures.

Given the specific phenotypic combination and the challenges of prospective recruitment in pediatric ophthalmology, the inclusion of 30 eyes was considered both scientifically meaningful and ethically appropriate for a pilot investigation. While the sample size does not allow for broad generalization, it provides sufficient power for an in-depth exploration of clinical and biochemical outcomes within a real-world clinical setting. The prospective nature of the study, together with the application of strict eligibility criteria and standardized follow-up protocols, contributes to strong internal validity and ensures methodological consistency. These features collectively establish a robust foundation for future multicenter trials aimed at validating and expanding these preliminary observations.

Demographic and baseline characteristics are reported in Table 1.

**Table 1:** Demographic characteristics and baseline values

Characteristic	Baseline value
Patients (n)	15
Treated eyes (n)	30
Age (years), mean ± SD	16.41 ± 2.36
Age range (years), min–max	12–18
Age inclusion criteria (years)	≥ 10 and ≤ 18
Sex, male	11 (73.3%)
Sex, female	4 (26.7%)
Ethnicity	Caucasian
Kmax (D), mean ± SD (95% CI)	55.00 ± 0.70 (95% CI: 54.75–55.25)
Keratoconus stage (Krumeich)	Stage II (100%)
CDVA (logMAR), mean ± SD (95% CI)	0.17 ± 0.04 (95% CI: 0.16–0.19)
OSDI, mean ± SD (95% CI)	24.5 ± 3.4 (95% CI: 23.3–25.7)
cBUT (seconds), mean ± SD (95% CI)	10.11 ± 0.84 (95% CI: 9.81–10.41)
MCT (μm), mean ± SD (95% CI)	457.0 ± 36.3 (95% CI: 444.0–470.0)
Tear MMP-9 (ng/mL, per patient, n = 15), mean ± SD (95% CI)	64.79 ± 30.27 (95% CI: 49.48–80.11)

Baseline values are reported as mean ± SD with 95% CI in parentheses when available. Kmax, CDVA, OSDI, cBUT and MCT baseline statistics were reconstructed from figures (per-eye analysis, n = 30). MMP-9 baseline statistics were calculated from patient-level data (n = 15). For OSDI, SE of the paired difference and 95% CI for the change versus baseline are provided in Supplementary Table S1

*SD* = standard deviation; *Kmax* = maximum keratometry; *CI* = confidence interval; *CDVA* = corrected distance visual acuity; *OSDI* = Ocular Surface Disease Index; *cBUT* = computerized non-invasive tear break-up time; *MCT *= minimum corneal thickness; *MMP-9* = matrix metalloproteinase-9

All eyes underwent bilateral sequential epi-off ACXL (Siena Cross-linking Center, Siena, Italy), first the worst eye, then the fellow eye after 1 month, using 9 mW/cm^2^ UVA for 10 min (total fluence 5.4 J/cm^2^). Protocol details are provided in Table 2.

**Table 2:** Epithelium-off accelerated corneal cross-linking (epi-off ACXL) protocol parameters

Parameter	Value
Treatment target	Ectasia stabilization
Thinnest point (µm)	≥ 400
Fluence (total) (J/cm^2^)	5.4
Soaking time (min)	10
Intensity (mW/cm^2^)	9
Irradiation time (min)	10
Epithelium status	Off
Chromophore	Riboflavin 0.1% (HPMC 1%, isotonic)
Light source	New KXL I (Glaukos-Avedro, USA)
Irradiation mode (interval)	Continuous

Epi-off ACXL at 9 mW/cm^2^ for 10 min (total fluence 5.4 J/cm^2^)

Exclusion criteria comprised a history of ocular trauma, contact lens wear, corneal opacities, infectious keratitis, or any previous ocular surgery. Patients who received topical ophthalmic pharmacologic therapy prior to baseline evaluation were also considered ineligible for study participation.

### KC progression criteria

KC progression was defined as any of the following changes documented over the preceding 6 months on serial examinations: increase in maximum keratometry (Kmax) ≥ 1.0 D on anterior corneal tomography obtained with a Scheimpflug-Placido system (Sirius, Costruzione Strumenti Oftalmici, Florence, Italy); reduction in minimum corneal thickness (MCT) ≥ 10 μm; worsening of visual acuity: decrease in uncorrected distance visual acuity (UDVA) or corrected distance visual acuity (CDVA) by ≥ 0.1 decimal units; increase in mean refractive spherical equivalent (MRSE) ≥ 0.50 D. Eligibility for ACXL additionally required MCT ≥ 400 μm (epithelium included) [23].

### Main outcome measures

The prespecified outcome measures included CDVA, Kmax, MCT, computerized non-invasive tear break-up time (cBUT), the OSD (0–100), and tear matrix metalloproteinase-9 (MMP-9) (Table 3).

**Table 3:** Metalloproteinase-9 (MMP-9) values (ng/mL)

Patient No	Baseline	6 months	12 months
1	54.8	39.0	35.4
2	41.7	15.8	9.1
3*	115.1*	46.1*	40.6*
4	62.9	22.8	18.0
5*	143.3*	42.8*	40.4*
6	39.5	9.8	7.4
7	46.1	1.5	0.2
8	39.7	6.9	0.6
9	50.5	3.3	0.4
10	89.9	27.7	22.3
11	45.4	12.9	11.3
12	77.8	34.0	27.2
13	66.7	24.9	18.8
14	51.3	9.2	6.5
15	47.2	6.6	4.1
Mean	64.79	20.22	16.15

^*^indicates vernal keratoconjunctivitis (VKC). Data are individual values

The OSDI was assessed at each study visit. Patients completed the questionnaire upon arrival, providing a standardized measure of dry eye symptoms and their impact on vision-related daily activities. The OSDI is a validated 12-item instrument with responses scored from 0 (“none of the time”) to 4 (“all of the time”), yielding a composite score ranging from 0 to 100. Higher scores indicate greater symptom severity. For statistical purposes, OSDI values were analyzed both as continuous and categorical variables according to established severity cutoffs [24]. In addition to the continuous score (primary analysis), OSDI severity was prespecified and categorized as normal (0–12), mild (13–22), moderate (23–32), and severe (≥ 33) [24]. The categorical analysis, presented in the results section, complements the continuous data. At baseline, OSDI severity was predominantly mild in 10 of 15 eyes (66.7%), moderate in 3 (20.0%), and severe in 2 (13.3%), the latter corresponding to the two cases with VKC.

Spectacle-CDVA was assessed at baseline and at 1, 3, 6, and 12 months using a standard Snellen chart under consistent photopic conditions. Results were recorded in decimal notation and converted to logMAR prior to statistical analysis.

Corneal topographic parameters, including Kmax and MCT, were assessed using a combined Scheimpflug–Placido tomography system (Sirius, CSO, Florence, Italy). Tear film stability was evaluated on the same platform by means of a non-contact videokeratoscopic protocol, and cBUT was expressed in seconds.

Tear MMP-9 concentration was quantified at baseline, 6 months, and 12 months using a point-of-care immunoassay system (i-ImmunDx™ Analyzer, Seinda Corporation, Guangzhou, China) [25]. Following tear collection with a micro-capillary fluid collector (MCFC), samples were loaded onto the reagent card, and three drops of assay buffer were added. Quantitative results were automatically generated by the analyzer after approximately 10 min. Pathological MMP-9 levels were defined as concentrations > 38.6 ng/mL in a tear volume of 2.2 μL (limit of detection ≤ 5 ng/mL) [25]. Tear samples were collected from the inferior tear meniscus under slit-lamp visualization, prior to fluorescein staining or any other diagnostic test, and without the use of topical anesthetic to minimize both reflex tearing and potential interference with tear biochemical composition. For consistency, tears were obtained from the more advanced eye, as determined by baseline Kmax, and the same eye was followed longitudinally throughout the study.

IVCM was performed using the Heidelberg Retina Tomograph II with the Rostock Cornea Module (HRT II-RCM, Heidelberg Engineering, Germany), which employs a 670-nm Helium–Neon laser source providing single-wavelength illumination with high contrast, about 1–2 μm lateral resolution, and 4–25 μm optical section thickness along the z-axis [26]. Examinations were conducted in a standardized sequence at baseline and at the 1-year follow-up visit.

IVCM was used as a qualitative exploratory outcome to document the presence and subsequent clearance of inflammatory cell infiltrates, since the study aimed to assess the anti-inflammatory effect of ACXL rather than quantify cell density.

### Surgical procedure

All treatments were performed at the Siena Cross-linking Center (Siena, Italy) by the same experienced surgeon (CM). The epi-off ACXL protocol was delivered using the New KXL I system (Glaukos, Burlington, MA, USA) at 9 mW/cm^2^ for a total fluence of 5.4 J/cm^2^. Procedures were performed sequentially (first one eye, then the fellow eye after 1 month) under identical parameters.

Topical anesthesia was achieved with 4% oxybuprocaine hydrochloride (1.6 mg/0.4 mL drops) instilled 10 min before treatment, following premedication with 2% pilocarpine administered 30 min prior to the procedure. After placement of a closed-valve eyelid speculum, the central 9 mm of corneal epithelium was gently removed using a blunt metal spatula.

A dextran-free 0.1% riboflavin isotonic solution containing hydroxyl-propyl methylcellulose (HPMC) (VibeX Rapid, Glaukos, Burlington, MA, USA) was instilled at one drop per minute for 10 min to allow corneal soaking prior to UVA exposure. Continuous UVA irradiation was then applied for 10 min at an irradiance of 9 mW/cm^2^, with two drops of riboflavin solution administered every 2.5 min throughout irradiation to maintain stromal saturation.

At the end of exposure, the corneal surface was rinsed with balanced saline solution (BSS) and medicated with preservative-free netilmicin plus dexamethasone and cyclopentolate eye drops. A therapeutic bandage contact lens was applied for 4 days. Following lens removal, fluorometholone 0.2% eye drops were prescribed three times daily and tapered over 30 days, along with a tear substitute containing 0.15 g hyaluronic acid sodium salt and 0.05 g Ginkgo biloba dry extract for 40 days.

### Statistical analysis

Statistical analyses were performed using Python (3.x) and R (4.x). Continuous variables are expressed as mean ± standard deviation (SD) with 95% confidence interval (CI). Corneal and visual outcomes (Kmax, CDVA, cBUT, OSDI, MCT; n = 30 eyes) were analyzed per eye, whereas tear MMP-9 was analyzed per patient (n = 15). Within-subject changes from baseline were assessed using paired t-tests, with Wilcoxon signed-rank tests used as sensitivity analyses when normality was uncertain. Effect sizes are reported as mean change (Δ) from baseline with 95% CI; for OSDI, standard error (SE) and 95% CI of Δ are also provided. Changes in the proportion of patients below the pathological MMP-9 threshold (38.6 ng/mL) were tested using a two-sided exact binomial test. All analyses were two-sided with α = 0.05, interpreted as exploratory without multiplicity adjustment.

## Results

CDVA showed a significant improvement over time following epi-off ACXL. Mean CDVA improved from 0.17 logMAR at baseline (≈ 0.67 decimal; 95% CI: 0.16–0.19 logMAR) to 0.20 logMAR at 1 month (≈ 0.63 decimal; 95% CI: 0.19–0.21 logMAR), followed by progressive recovery to 0.15 logMAR at 3 months (≈ 0.71 decimal; 95% CI: 0.14–0.16 logMAR). A significant improvement was observed at 6 months (0.09 logMAR; ≈ 0.81 decimal; 95% CI: 0.08–0.10 logMAR; *P* < 0.001), which remained stable up to 12 months (0.09–0.10 logMAR range).

**Fig. 1:**
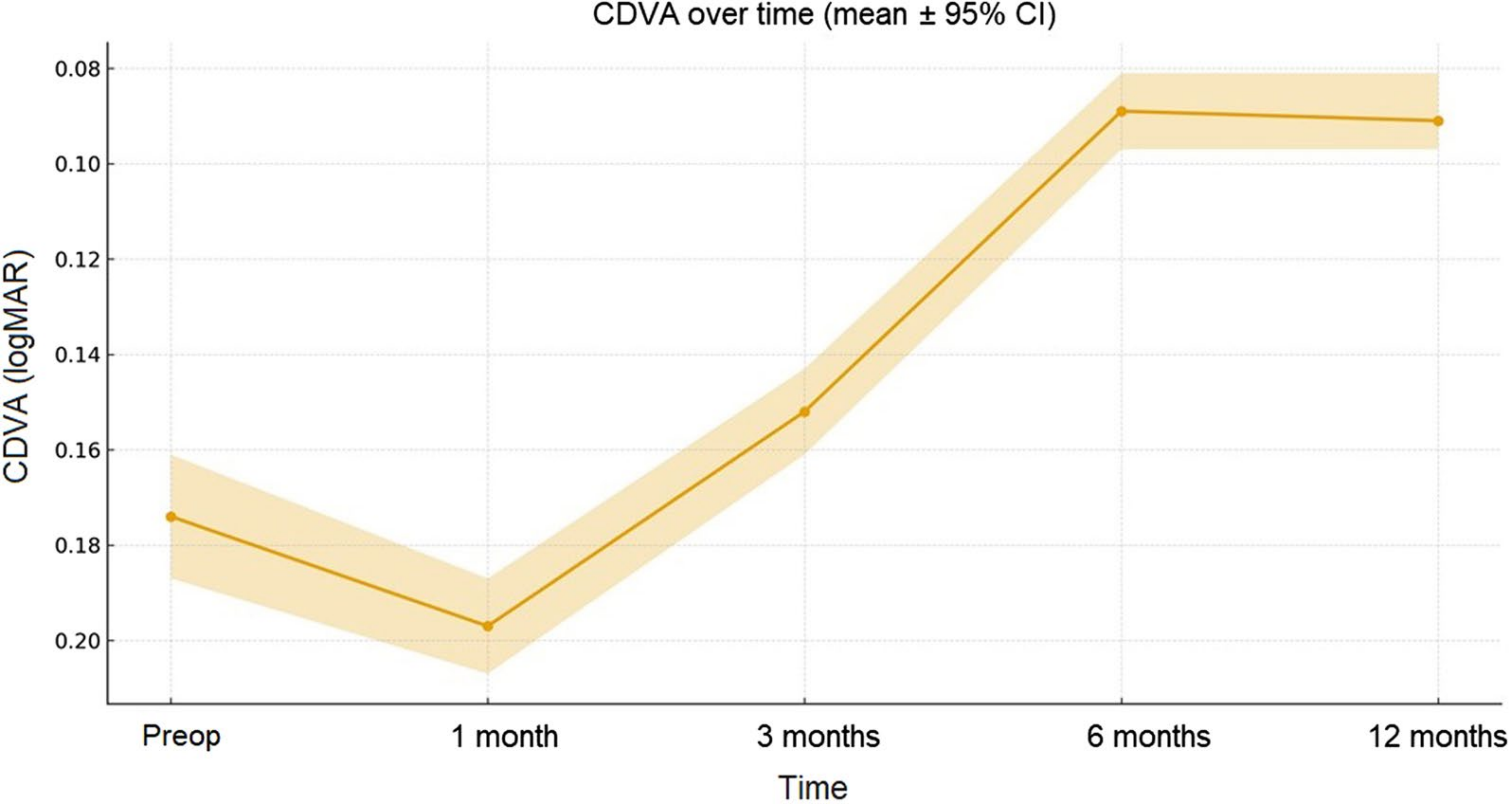
Temporal changes in corrected distance visual acuity (CDVA), expressed in logMAR, following epi-off accelerated corneal cross-linking (ACXL; 9 mW/cm^2^, 5.4 J/cm^2^). Data are shown as mean ± 95% confidence interval (CI) at baseline and at 1-, 3-, 6-, and 12-month follow-up (per-eye analysis, n = 30). Visual acuity measurements were converted from decimal notation to logMAR prior to statistical analysis. CDVA improved significantly from baseline, reaching 0.09 logMAR at 12 months (≈ 0.81 decimal; 95% CI: 0.10–0.08 logMAR; *P* < 0.001)

Overall, CDVA improvement from baseline through the 12-month follow-up was statistically significant (*P* < 0.001), as illustrated in Fig. 1.

**Fig. 2:**
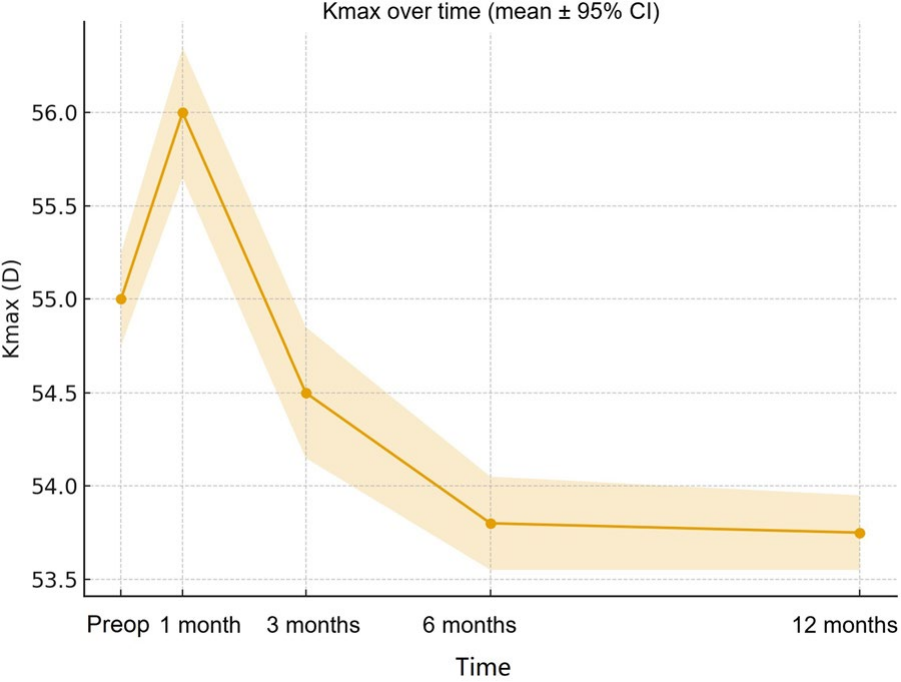
Temporal changes in maximum keratometry (Kmax) after epi-off accelerated corneal cross-linking (ACXL; 9 mW/cm^2^, 5.4 J/cm^2^). Data are expressed as mean ± 95% confidence interval (CI) at baseline and at 1-, 3-, 6-, and 12-month follow-up (per-eye analysis, n = 30 eyes). A progressive and statistically significant flattening was observed from baseline to 12 months (mean at 12 months: 53.75 D; 95% CI: 53.55–53.95 D; *P* < 0.001, paired t-test), indicating stabilization of corneal ectasia

Kmax showed a consistent flattening over time, decreasing to 53.75 D at 12 months (95% CI: 53.55–53.95 D; *P* < 0.001), indicating stabilization of corneal ectasia (Fig. 2). Baseline Kmax averaged 55.00 D (95% CI: 54.75–55.25 D), with a mean change (Δ) of − 1.25 D at 12 months (95% CI for Δ: − 1.57 to − 0.93 D).

**Fig. 3:**
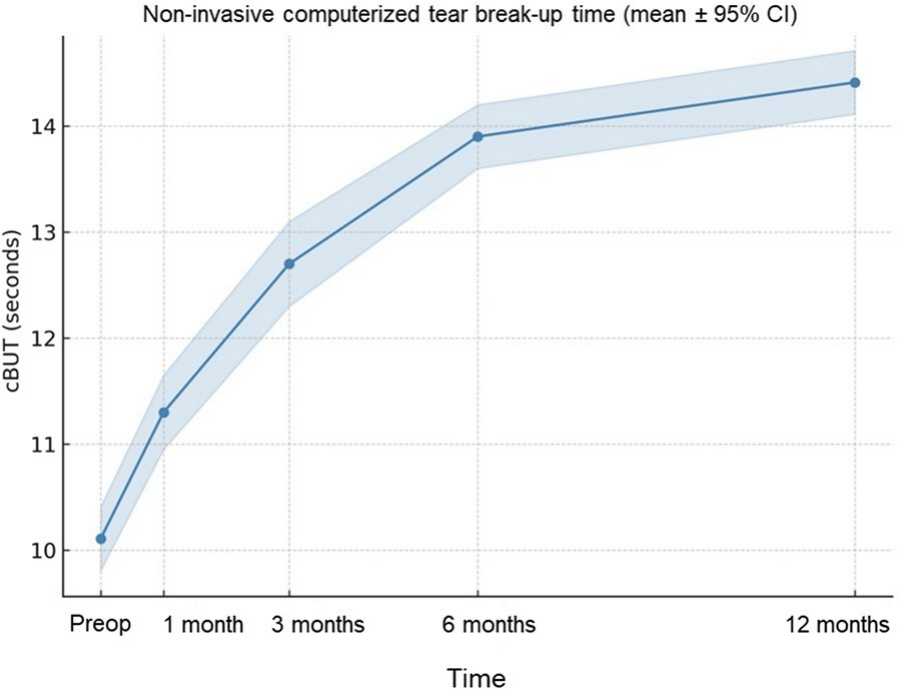
Temporal changes in computerized non-invasive tear break-up time (cBUT) after epi-off accelerated corneal cross-linking (ACXL; 9 mW/cm^2^, 5.4 J/cm^2^). Data are shown as mean ± 95% confidence interval (CI) at baseline and at 1-, 3-, 6-, and 12-month follow-up (per-eye analysis, n = 30 eyes). cBUT increased significantly over time (12 months: 14.41 s; 95% CI: 14.11–14.71 s; *P* < 0.01, paired t-test), indicating improved tear film stability

cBUT increased from 10.11 s at baseline (95% CI: 9.81–10.41 s) to 14.41 s at 12 months (95% CI: 14.11–14.71 s; *P* < 0.01), indicating an improvement in ocular surface stability (Fig. 3).

**Fig. 4:**
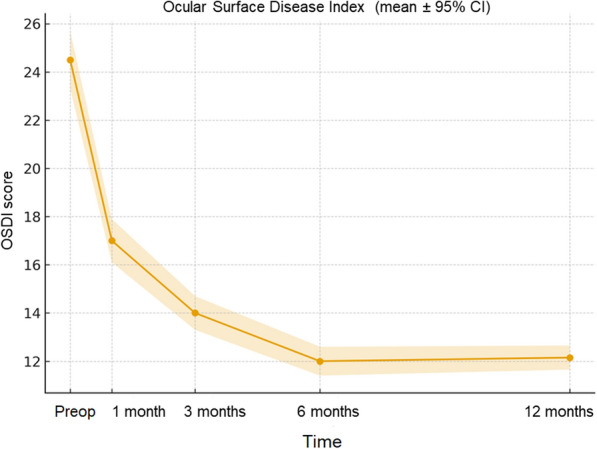
Temporal changes in Ocular Surface Disease Index (OSDI) scores after epi-off accelerated corneal cross-linking (ACXL; 9 mW/cm^2^, 5.4 J/cm^2^). Data are reported as mean ± 95% confidence interval (CI) at baseline and at 1-, 3-, 6-, and 12-month follow-up (per-eye analysis, n = 30 eyes). A continuous decrease in OSDI scores was observed through 12 months (mean at 12 months: 12.15; 95% CI: 11.65–12.65), reflecting improved ocular surface comfort

OSDI scores decreased progressively over time, reaching a mean of 12.15 at 12 months (95% CI: 11.65–12.65), comparable to the values observed at 6 months (Fig. 4). The mean change (Δ) from baseline at 12 months was − 12.35 (SE of paired difference ≈ 0.66; 95% CI for Δ: − 13.65 to − 11.05), indicating a sustained improvement in subjective ocular surface symptoms.

**Fig. 5:**
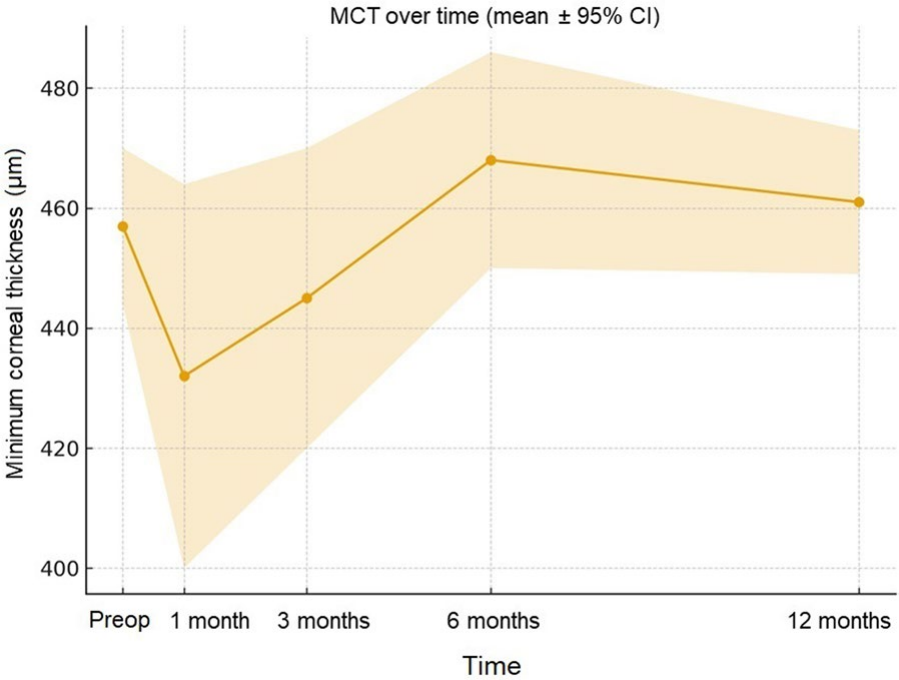
Temporal changes in minimum corneal thickness (MCT) after epi-off accelerated corneal cross-linking (ACXL; 9 mW/cm^2^, 5.4 J/cm^2^). Data are presented as mean ± 95% confidence interval (CI) at baseline and at 1-, 3-, 6-, and 12-month follow-up (per-eye analysis, n = 30 eyes). A transient reduction at 1 month was followed by recovery and stabilization through 12 months

MCT exhibited a transient decrease at 1 month, followed by recovery and subsequent stabilization through 12 months (Fig. 5). Baseline MCT averaged 457 µm (95% CI: 444–470 µm) and measured 461 µm at 12 months, consistent with long-term structural stability.

**Fig. 6:**
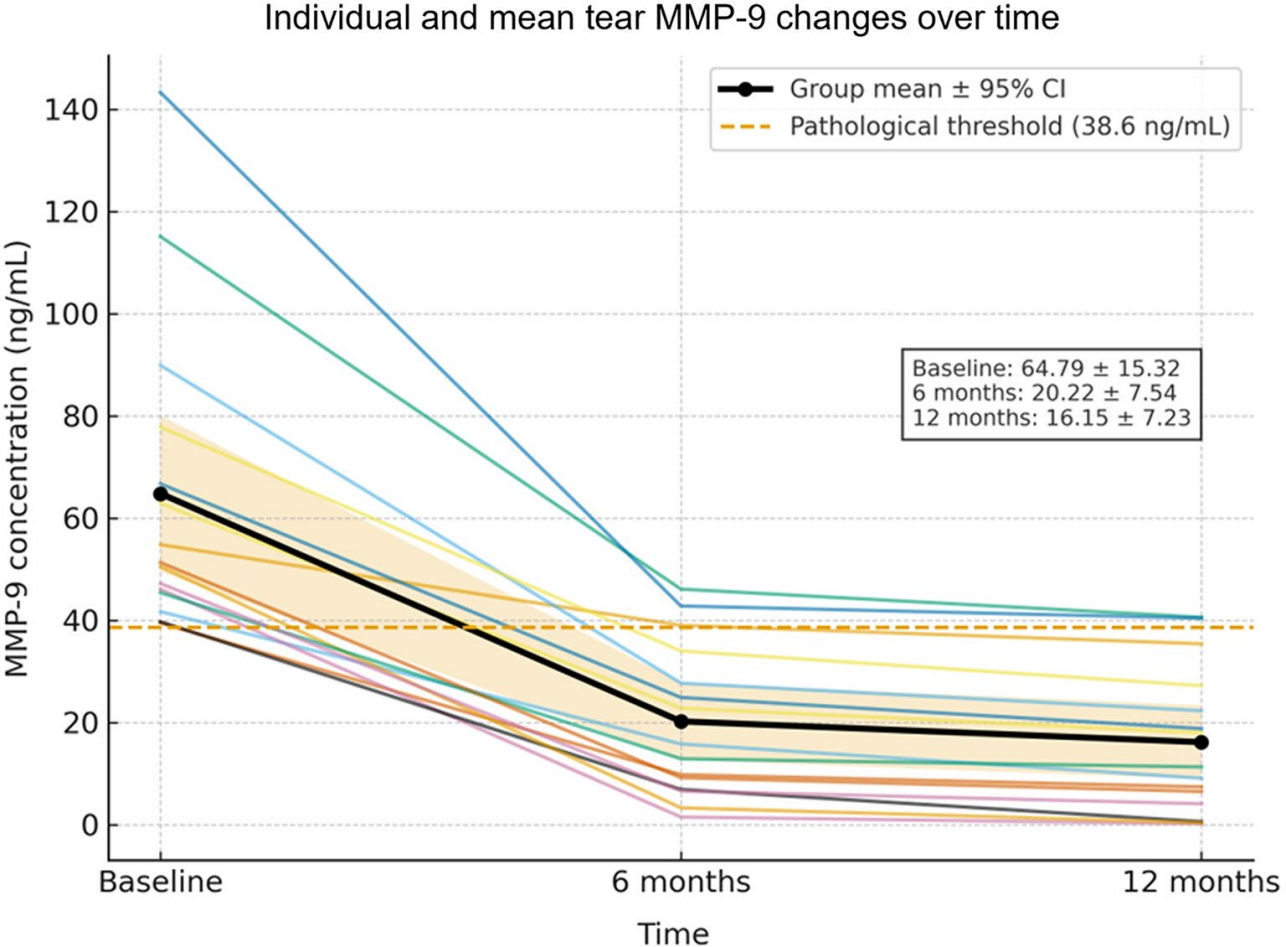
Temporal changes in tear matrix metalloproteinase-9 (MMP-9) concentration (ng/mL) after epi-off accelerated corneal cross-linking (ACXL; 9 mW/cm^2^, 5.4 J/cm^2^). Individual MMP-9 trajectories (“spaghetti plot”) from baseline to 6 and 12 months (per-patient analysis, n = 15), with superimposed group mean ± 95% confidence interval (CI) and the pathological threshold (38.6 ng/mL, dashed line). A marked and statistically significant reduction was observed from baseline to 12 months (*P* < 0.0001, paired t-test), with mean concentrations falling below the pathological threshold of 38.6 ng/mL in most eyes

At both 6 and 12 months, tear MMP-9 concentrations (per-patient analysis, n = 15) were significantly reduced compared with baseline (*P* < 0.0001, paired t-test). The proportion of patients below the pathological threshold of 38.6 ng/mL increased from 0.0% at baseline to 80.0% at 6 months and 86.7% at 12 months (*P* < 0.0001 at both time points; exact binomial test). A further significant reduction in mean MMP-9 concentration was observed between 6 and 12 months (Fig. 6). Notably, the two patients with concomitant VKC, despite elevated MMP-9 levels above the pathological threshold, both exhibited marked individual reductions from baseline accompanied by clear clinical improvement, including decreased photophobia, conjunctival hyperemia, and tearing.

**Fig. 7:**
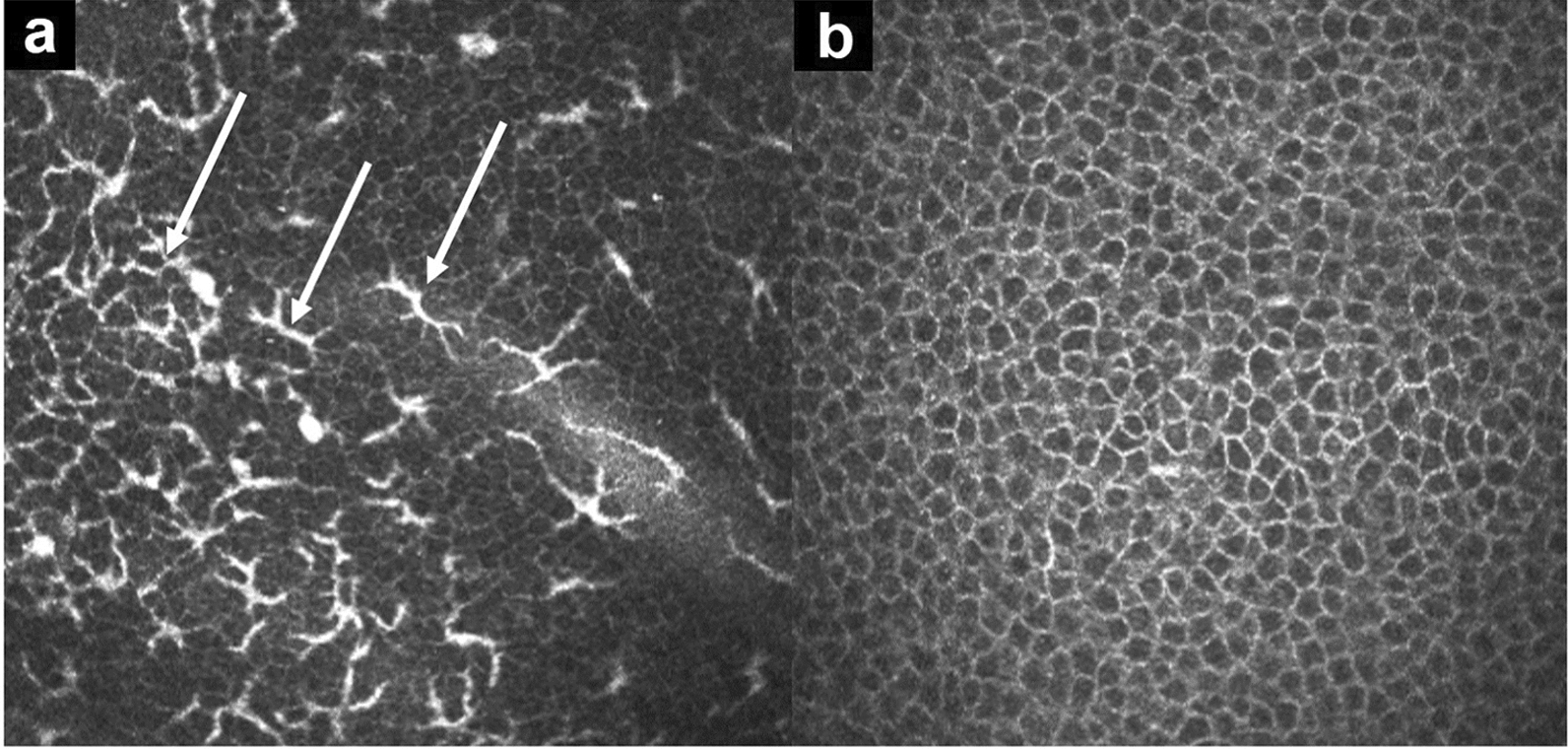
Representative in vivo confocal microscopy (IVCM) images of the basal corneal epithelium. Scan depth 40–45 µm. **a** Preoperative image showing multiple hyperreflective dendritic Langerhans cells within the basal epithelial layer, consistent with active ocular-surface inflammation; **b** One month after epi-off accelerated corneal cross-linking (ACXL), Langerhans cells are no longer detectable, and epithelial architecture appears normalized, indicating effective clearance of inflammatory cells

**Fig. 8:**
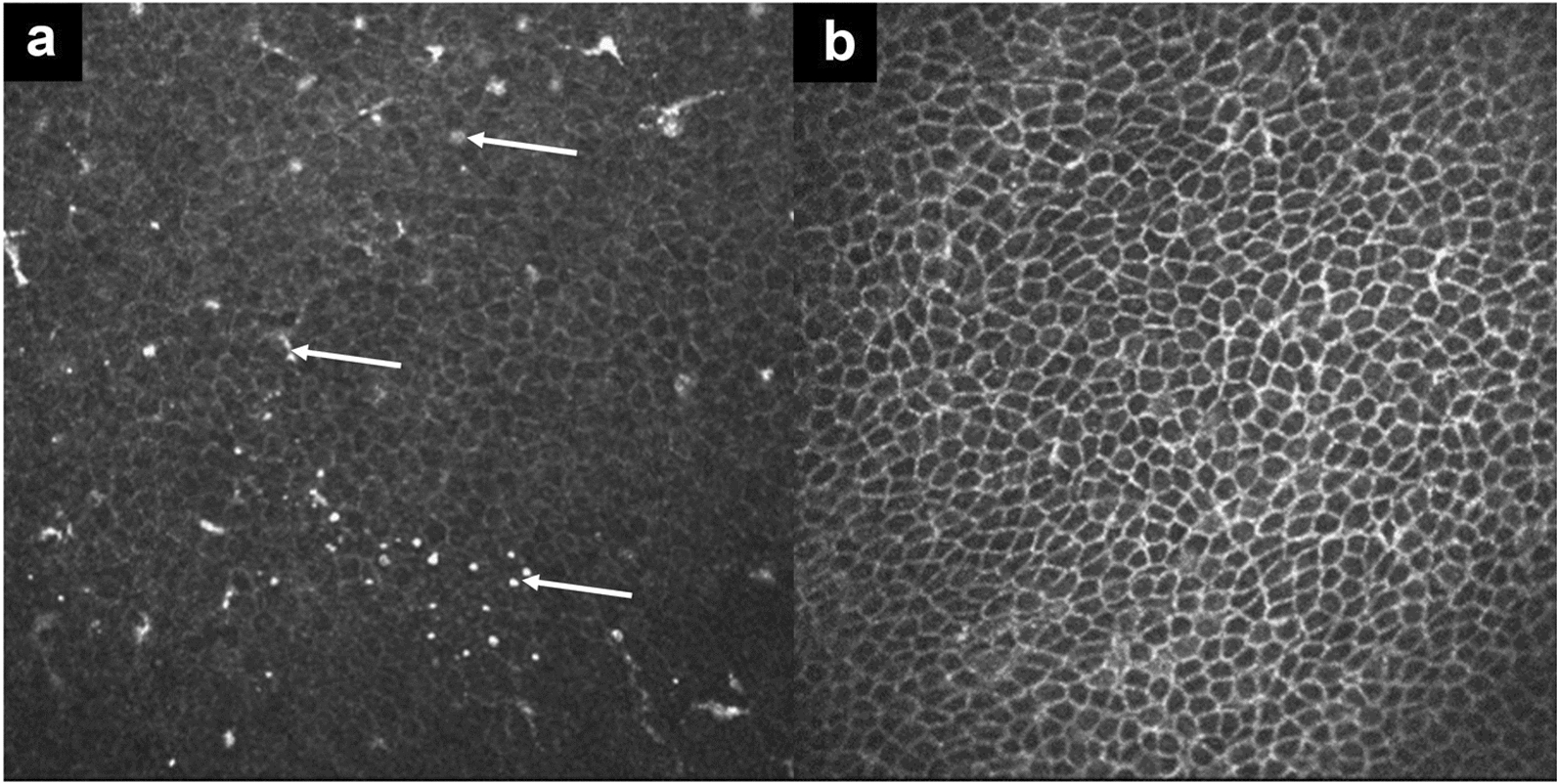
Representative in vivo confocal microscopy (IVCM) images of the basal corneal epithelium in a pediatric patient with progressive keratoconus, allergic ocular surface disease, and dry eye. Scan depth 40–45 µm. **a** Preoperative scan showing an evident inflammatory infiltrate composed of bright hyperreflective micro-particles consistent with polymorphonuclear leukocytes and lymphocytes within the basal corneal epithelium; **b** One month after epi-off accelerated corneal cross-linking (ACXL), inflammatory cells are no longer detectable

Preoperative IVCM in patients with VKC, showed the presence of Langerhans dendritic cells at the level of the basal corneal epithelium (Fig. 7a, white arrows, scan depth ~ 45 µm). One month after epi-off ACXL (9 mW/cm^2^, 5.4 J/cm^2^), IVCM scan demonstrated a complete disappearance of Langerhans cells (Fig. 7b). In a patient with allergy and elevated tear MMP-9, preoperative qualitative IVCM scan of basal corneal epithelium showed bright microparticles compatible with polymorphonuclear granulocytes/lymphocytes (Fig. 8a, white arrows, scan depth ~ 50 µm). One month after ACXL, the epithelial mosaic appeared regular with well-defined cell borders and no inflammatory cells (Fig. 8b).

No permanent adverse events occurred. Transient postoperative effects included delayed epithelial healing (2/30, 6.7%), sterile corneal infiltrates (2/30, 6.7%), pain > 48 h [3/30, 10.0%; resolved within 72 h with non-steroidal anti-inflammatory drugs (NSAIDs)], and photophobia/punctate keratitis (5/30, 16.7%; resolved within 7 days with topical steroids and lubricants); all resolved without sequelae (Table 4).

**Table 4:** Adverse events

Event	Eyes affected, n/N (%)	Notes
Any adverse event	0/30 (0.0%)	–
Intraoperative complication	0/30 (0.0%)	–
Delayed epithelial healing (> 7 days)	2/30 (6.7%)	–
Infectious keratitis	0/30 (0.0%)	–
Sterile corneal infiltrates	2/30 (6.7%)	–
Corneal haze (≥ Grade 1)	0/30 (0.0%)	–
Corneal melt/perforation	0/30 (0.0%)	–
Steroid-induced IOP rise (≥ 10 mmHg)	0/30 (0.0%)	–
Persistent pain > 48 h	3/30 (10.0%)	Resolved within 72 h with NSAIDs
Photophobia/punctate keratitis	5/30 (16.7%)	Resolved within 7 days with topical steroids
Other	0/30 (0.0%)	–

Counts are per treated eye (n = 30). Percentages are calculated as n/N × 100% and rounded to one decimal place. All events are resolved without sequelae

*NSAIDs* = non-steroidal anti-inflammatory drugs

## Discussion

In this prospective pilot study involving pediatric eye-rubbers patients with progressive KC suffering from allergy, dry eye symptoms, and elevated tear MMP-9 levels, epi-off ACXL was associated with stabilization of corneal ectasia (Kmax), improvement in visual function (CDVA) and tear film stability (cBUT), reduction in subjective symptom burden (OSDI), and a marked decline in tear MMP-9 concentration, demonstrating a favorable safety and tolerability profile. Historically, concerns have been raised that corneal cross-linking (CXL) might exacerbate dry eye symptoms owing to transient epithelial trauma and corneal nerve disruption [27]. However, Kontadakis et al. [28] reported that despite a temporary reduction in corneal innervation and sensitivity for up to 6 months after treatment, basic tear secretion and stability remained largely unaffected, which is consistent with the postoperative symptomatic improvement observed in our series [28].

Similarly, Taneri et al. [29] reported no detrimental effect on objective dry eye parameters at 3 to 6 months, while Wang et al. documented improved tear function outcomes following CXL [30]. Taken together, these findings and ours support the view that CXL does not adversely affect, and may even enhance, ocular surface status in selected patients.

A coherent biological framework links riboflavin/UVA photochemistry and stromal biomechanics to ocular surface photobiomodulation. In this context, epi-off ACXL demonstrated a reduction in tear MMP-9 expression and an improvement in cBUT in our series, consistent with prior reports [20–22].

Benefits likely stem from reactive oxygen species (ROS)-mediated cross-link formation [31–33] and photobiomodulation on the ocular surface [24], with synergistic riboflavin–UVA interactions [31]. Riboflavin exhibits antioxidant, anti-inflammatory [nuclear factor-kappa B (NF-κB) inhibition; interleukin (IL)-1β, tumor necrosis factor (TNF)-α, IL-6], inflammasome downregulation (caspase-1, NLRP3), and anti-lymphangiogenic effects [vascular endothelial growth factors A/C (VEGF-A/C), MMP-9], while supporting mitochondrial function and neuroregeneration via flavoenzymes [32–34]. When UVA-activated, these actions are amplified: antivasoproliferative effects [35–37], keratocyte and inflammatory-cell apoptosis [26], and MMP-9 downregulation [22], collectively improving epithelial healing and tear film stability.

**Fig. 9:**
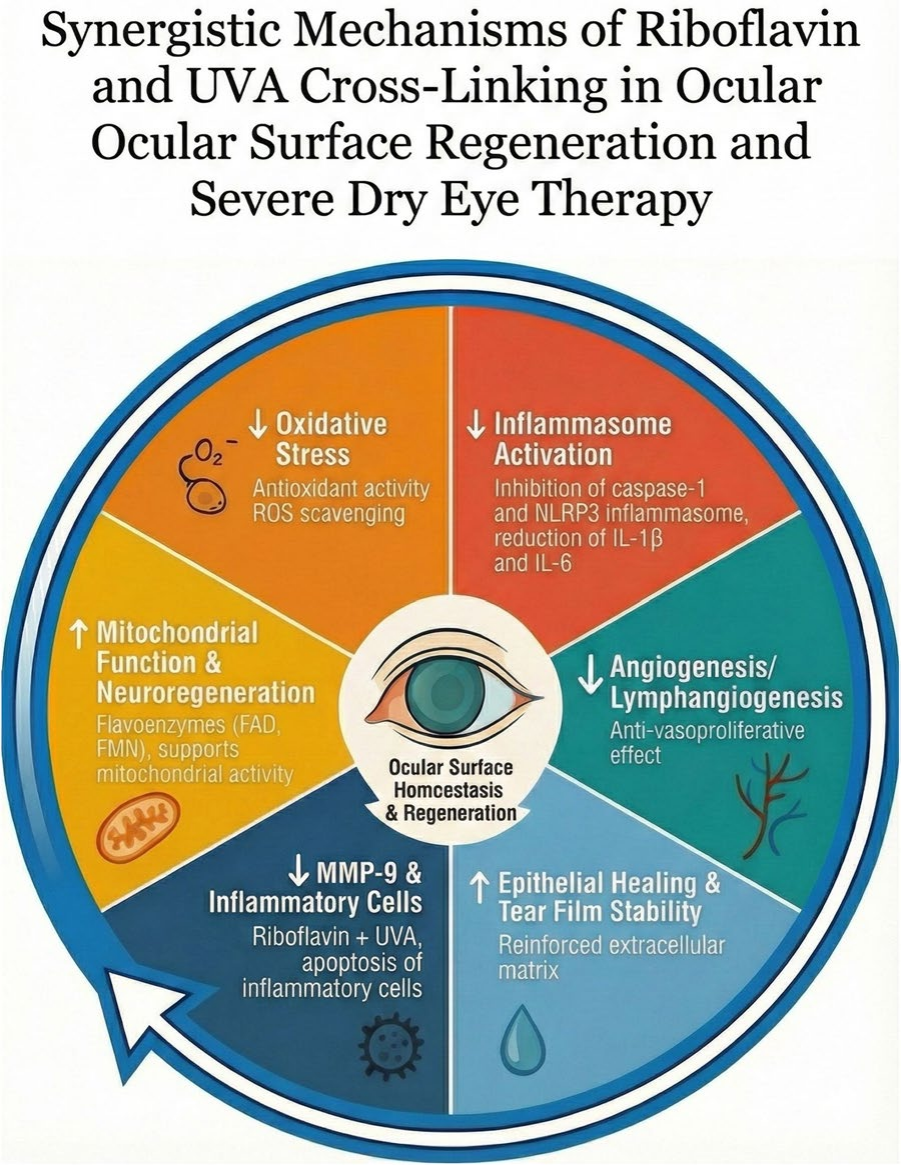
Riboflavin/UVA synergy combines biomechanical reinforcement with anti-proteolytic, photobiomodulatory, and neuromodulatory effects, stabilizing keratoconus and restoring ocular surface homeostasis. Epi-off accelerated corneal cross-linking (ACXL; 9 mW/cm^2^, 5.4 J/cm^2^) exerts synergistic biomechanical, anti-proteolytic, photobiomodulatory, and neuromodulatory effects in pediatric keratoconus with allergic ocular surface disease and elevated tear MMP-9. UVA-activated riboflavin strengthens corneal stroma, downregulates MMP-9, and restores ocular surface homeostasis through combined biomechanical and anti-inflammatory mechanisms

These interrelated pathways support a realistic “Virtuous Circle” model proposed by Mazzotta (Fig. 9), whereby riboflavin/UVA cross-linking reduces oxidative stress and inflammation, limits hemangiogenesis and lymphangiogenesis, enhances epithelial/stromal repair, and stabilizes the tear film, ultimately restoring ocular surface homeostasis. Importantly, biomarker-guided patient stratification is gaining traction—ocular surface inflammation has been implicated in KC progression [38]; a pathogenic triad comprising allergy, eye rubbing, and elevated IgE–MMP-9 levels has been proposed [39]; and MMP-9 positive allergic patients have been shown to be at increased risk of progression, as documented by Mazzotta et al. [11]. In line with Recalde et al. [22], our sustained MMP-9 reduction supports a genuine ACXL-induced anti-inflammatory signal, also in VKC patients.

The neuromodulatory effects of ACXL may also play a contributory role: temporary disruption of the sub-basal nerve plexus, followed by gradual reinnervation [26, 31], may attenuate neurogenic inflammation and interrupt the itch–rub–inflammation cycle which ultimately promotes behavioral stabilization through reduced eye-rubbing [13, 40]. Although early postoperative use of topical steroids and lubricants could transiently influence ocular surface parameters, the sustained MMP-9 reduction of up to 12 months strongly suggests a long-term anti-inflammatory effect of ACXL rather than a medication-related response. Clinically, patients reported reduced photophobia, ocular redness, and reflex tearing, along with greater ease in maintaining fixation during corneal tomography, all consistent with decreased inflammation and improved ocular surface homeostasis. IVCM further supported these findings by demonstrating postoperative resolution of inflammatory cell infiltrates, consistent with the observations previously reported by Ferrini et al. [41].

These findings are particularly relevant in the pediatric population, where KC tends to be more aggressive [42, 43] and is frequently associated with allergic ocular surface inflammation. The observed reduction in tear MMP-9 levels and inflammatory activity suggest a beneficial anti-inflammatory effect of ACXL on the ocular surface, beyond its known biomechanical stiffening action. This aligns with our previous evidence that elevated MMP-9 contributes to KC progression and worsens ocular surface dryness, highlighting the potential of ACXL to modulate both disease activity and ocular surface homeostasis in pediatric, allergic and VKC associated KC [44, 45]. Collectively, these results support a dual-action rationale for epi-off ACXL, combining corneal biomechanical stabilization with modulation of ocular surface inflammation. This evidence provides the basis for future controlled trials focused on dry eye outcomes and biomarker-driven (e.g., MMP-9) patient selection to explore ACXL as an adjunctive therapy in DED both in children and adults, beyond KC.

Alongside the hypothesized neuromodulatory, photobiomodulatory, and anti-inflammatory mechanisms suggested by the sustained reduction in tear MMP-9 levels and the long-term improvement of ocular surface parameters, it is also appropriate to acknowledge the positive contribution of CXL-induced corneal geometric normalization. The associated decrease in corneal curvature may indeed enhance tear film stability and partially account for the clinical improvements reported by patients. However, within the context of our findings, these geometric effects appear to play a secondary role when compared with the more central and enduring biological impact of accelerated CXL on ocular surface homeostasis. The long-term improvements documented in the absence of continued pharmacological therapy further support the notion that CXL may influence the ocular surface microenvironment through a broader and more durable set of mechanisms than stromal stiffening alone.

Furthermore, although patients with chronic ocular surface alterations often experience recurrence of symptoms after tapering topical steroids, our cohort showed a progressive and sustained improvement well beyond the immediate postoperative period. In particular, several children, most notably those with VKC, demonstrated a remarkable functional recovery during the year following CXL. Symptoms such as photophobia, reflex tearing, and the inability to keep the eye open during examinations, which had previously limited even basic imaging procedures, were substantially reduced. This durable restoration of ocular surface comfort, rarely observed after short steroid courses, supports the hypothesis that CXL may promote a long-term stabilization of ocular surface homeostasis extending well beyond its biomechanical effects. Such a pattern appears more consistent with a sustained, CXL-mediated rebalancing of the ocular surface microenvironment than with a transient pharmacologic response. Although the present pilot study does not allow extrapolation to non-ectatic corneas, it provides preliminary evidence that CXL may exert combined biomechanical and biological benefits capable of improving ocular surface stability. These findings, while initial, highlight a potential broader therapeutic relevance of CXL that warrants further exploration through preclinical models and rigorously designed clinical studies.

Limitations of this study include its single-center design, single-arm study with a relatively small sample size, and lack of a control group. Nevertheless, the strict eligibility criteria and prospective follow-up strengthen internal validity and provide a solid foundation for future multicenter controlled investigations.

## Conclusions

This prospective pilot study shows that epi-off ACXL offers meaningful clinical benefits in pediatric patients with progressive KC and allergic ocular surface disease. ACXL stabilized ectatic progression and produced sustained improvements in tear film stability, symptoms, and tear MMP-9 levels, indicating biological effects that extend beyond corneal stiffening.

The consistent reduction in inflammatory activity, especially in children with VKC, suggests that ACXL may help interrupt the itch–rub–inflammation cycle and support long-term ocular surface homeostasis. Despite the study’s single-arm design and limited sample size, these findings provide compelling preliminary evidence for biomarker-guided patient selection, particularly in MMP-9 positive phenotypes.

Larger, controlled trials are needed to validate these results and to further explore the dual biomechanical and anti-inflammatory role of ACXL, as well as its potential broader relevance in ocular surface disease beyond KC.

## Supplementary Information

Additional file 1.

## Data Availability

The datasets generated and analysed during the current study are not publicly available due to restrictions related to patient privacy and pediatric data protection, but are available from the corresponding author on reasonable request.
