# Supplementary material for: Anti-inflammatory and dry eye benefits of accelerated epi-off corneal cross-linking in pediatric keratoconus with allergic ocular surface disease and elevated MMP-9
Source: Eye Vis (Lond). 2026 Jan 2;13:2. doi: 10.1186/s40662-025-00469-7 (PMC12763881; doi:10.1186/s40662-025-00469-7)
Supplement: Supplementary file 1 — Additional file 1. [file 40662_2025_469_MOESM1_ESM.docx]

**Supplementary Table S1. Mean change from baseline (Δ), SE(Δ), and 95% CI for Δ.**

| **Outcome** | **Δ timepoint** | **Baseline mean** | **Follow-up mean** | **Mean change (Δ)** | **SE (Δ)** | **95% CI for Δ (lower)** | **95% CI for Δ (upper)** |
| --- | --- | --- | --- | --- | --- | --- | --- |
| Kmax (D) | 1 month | 55.0 | 56.0 | 1.0 | 0.22 | 0.57 | 1.43 |
| Kmax (D) | 3 months | 55.0 | 54.5 | −0.5 | 0.22 | −0.93 | −0.07 |
| Kmax (D) | 6 months | 55.0 | 53.8 | −1.2 | 0.18 | −1.55 | −0.85 |
| Kmax (D) | 12 months | 55.0 | 53.75 | −1.25 | 0.16 | −1.57 | −0.93 |
| OSDI (score) | 1 month | 24.5 | 17.0 | −7.5 | 0.77 | −9.0 | −6.0 |
| OSDI (score) | 3 months | 24.5 | 14.0 | −10.5 | 0.71 | −11.89 | −9.11 |
| OSDI (score) | 6 months | 24.5 | 12.0 | −12.5 | 0.68 | −13.84 | −11.16 |
| OSDI (score) | 12 months | 24.5 | 12.15 | −12.35 | 0.66 | −13.65 | −11.05 |

SE = standard error; CI = confidence interval; Kmax = maximum keratometry; OSDI = Ocular Surface Disease Index; Δ = follow-up minus baseline.

SE(Δ) computed conservatively as √(SE_baseline² + SE_follow-up²), deriving SE from 95% CI half-widths (CI = ±1.96×SE). Values were reconstructed from figures; 12-month anchors were confirmed in-text. For OSDI, the reviewer requested CI for the difference and standard errors; these SE/CI are provided here.
